# Development of mortality prediction model in the elderly hospitalized AKI patients

**DOI:** 10.1038/s41598-021-94271-9

**Published:** 2021-07-26

**Authors:** Jing-Cheng Peng, Ting Wu, Xi Wu, Ping Yan, Yi-Xin Kang, Yu Liu, Ning-Ya Zhang, Qian Liu, Hong-Shen Wang, Ying-Hao Deng, Mei Wang, Xiao-Qin Luo, Shao-Bin Duan

**Affiliations:** 1 Hunan Key Laboratory of Kidney Disease and Blood Purification, Department of Nephrology, The Second Xiangya Hospital of Central South University, Changsha, 410011 Hunan China; 2 Information Center, The Second Xiangya Hospital of Central South University, Changsha, 410011 Hunan China

**Keywords:** Acute kidney injury, Risk factors

## Abstract

Acute kidney injury (AKI) correlates with increased health-care costs and poor outcomes in older adults. However, there is no good scoring system to predict mortality within 30-day, 1-year after AKI in older adults. We performed a retrospective analysis screening data of 53,944 hospitalized elderly patients (age > 65 years) from multi-centers in China. 944 patients with AKI (acute kidney disease) were included and followed up for 1 year. Multivariable regression analysis was used for developing scoring models in the test group (a randomly 70% of all the patients). The established models have been verified in the validation group (a randomly 30% of all the patients). Model 1 that consisted of the risk factors for death within 30 days after AKI had accurate discrimination (The area under the receiver operating characteristic curves, AUROC: 0.90 (95% CI 0.875–0.932)) in the test group, and performed well in the validation groups (AUROC: 0.907 (95% CI 0.865–0.949)). The scoring formula of all-cause death within 1 year (model 2) is a seven-variable model including AKI type, solid tumor, renal replacement therapy, acute myocardial infarction, mechanical ventilation, the number of organ failures, and proteinuria. The area under the receiver operating characteristic (AUROC) curves of model 2 was > 0.80 both in the test and validation groups. Our newly established risk models can well predict the risk of all-cause death in older hospitalized AKI patients within 30 days or 1 year.

## Introduction

Acute kidney injury (AKI) is one of the most common clinical syndrome in intensive care unit (ICU), which carries an increased cost, longer duration of hospitalization as well as a higher risk of mortality and morbidity^1^. According to the 2012 Kidney Disease: Improving Global Outcomes (KDIGO) guidelines^2^, AKI has been diagnosed and classified by either a rise in creatinine or a decrease in urine output. In hospitalized adult patients, the overall incidence of all forms of AKI is 3.0% to 18.3%^3^, and it reaches up to 30–60% in critically ill patients^4–6^. Some studies with > 10,000 patients reported that the long-term mortality rate of patients with AKI was 11.8–29.8%^7–9^. Recently, a published study reported that 46% of COVID-19 adult patients hospitalized in the Mount Sinai Health System had AKI and the median age of AKI patients was significantly higher than the non-AKI (71 VS 63 years, P < 0.001)^10^. The incidence and mortality of AKI in elderly people (age ≥ 65) was higher than that in younger patients due to “kidney aging”, polypharmacy and comorbidities^11^, which bring large attention to the disease progression of AKI in elderly patients.

Currently, the lack of favorable prognosis in AKI may be due to delayed clinical intervention. What clinicians do not know is that whether patients have an adverse prognosis and involve a timely and effective treatment. Some prognosis prediction systems such as Acute Physiology and Chronic Health Evaluation II (APACHE II)^12^ and Sequential Organ Failure Assessment (SOFA)^13^ that can predict mortality of AKI patients to some extent are often used in the ICU patients with multiple organ dysfunction (MODS). In 2016, we described a new scoring model to predict mortality in hospitalized patients with AKI^14^. The population is aging, and a strong relationship between age and increased mortality of AKI is clear^15^. Nowadays, although some studies have investigated the risk factors for predicting the prognosis of AKI in elderly patients^16^, an excellent model that can predict the mortality of elderly hospitalized AKI patients is essential. We study the incidence of AKI and risk factors of death within 30 days or 1 year in the elderly hospitalized patients and establish two risk models for predicting 30-day or 1-year all-cause death of AKI in the elderly. The present study aimed to understand and assess the prognosis of elderly AKI patients by using a practical prediction model that was built on clinical and laboratory data. We hope the new scoring models can facilitate the clinical individualized treatment and improve the prognosis of elderly AKI patients.

## Materials and methods

### Study population

**Figure 1:**
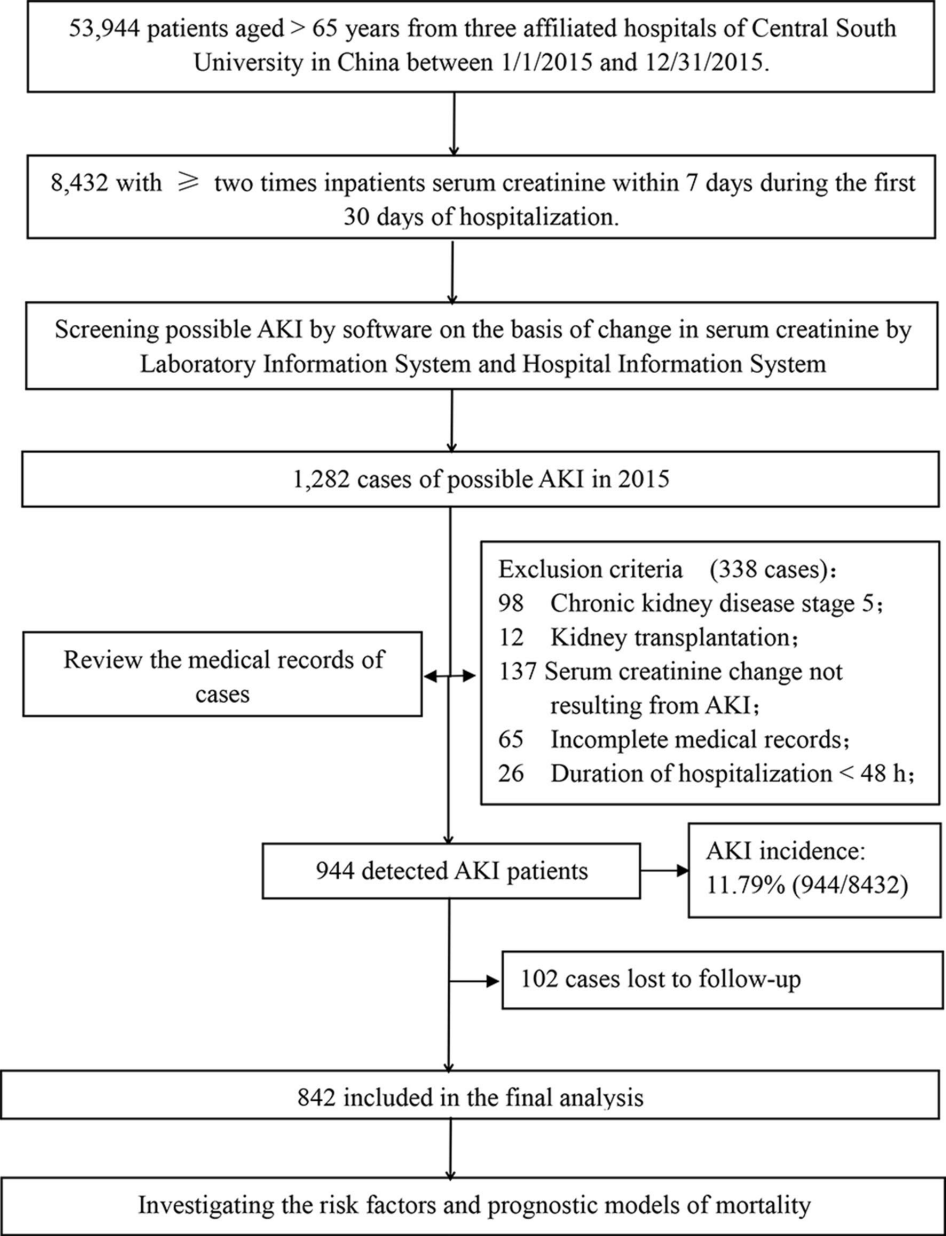
Study flow chart. AKI, acute kidney injury.

We conducted a retrospective cohort study screening data of 53,944 hospitalized older adults (older than 65 years of age) admitted to one of three hospitals in China (Xiangya hospital, Second Xiangya Hospital, and Third Xiangya Hospital), and patients were selected from January of 2015 to December of 2015. There were 19,445 patients from the First Xiangya Hospital, 24,057 patients from the Second Xiangya Hospital, and 10,442 patients from the Third Xiangya Hospital. According to the 2012 KDIGO criteria of AKI^2^, the changes of the patient's serum creatinine were screened by the Hospital Laboratory Information System to identify suspected AKI patients, if they had at least two inpatient serum creatinine (SCr) tests within a 7-day window during their first 30 days of hospitalization. Furthermore, we examined the medical records of the elderly patients with suspected AKI again to confirm the diagnosis of AKI. Our exclusion criteria were as follows: (1) patients diagnosed as ESRD (eGFR < 15 ml/min/1.72 m^2^) or renal transplantation before AKI diagnosis; (2) the SCr change not resulting from AKI (eg. SCr decrease attributed to limb amputation); (3) the duration of hospitalization < 48 h; (4) incomplete medical records. A total of 994 AKI patients were enrolled and followed-up and 842 AKI patients were followed up to the endpoints of the study or the endpoints were not happening before the final follow-up data. The study protocol was approved by the Medical Ethics Committee of the Second Xiangya Hospital of Central South University. All the methods in the present study were performed in accordance with guidelines of the Declaration of Helsinki. The informed consent was not signed by the patients because this study is retrospective. The study profile was shown in Fig. 1.

### Data collection and definition

Baseline demographic, clinical and laboratory data were obtained from the electronic medical record system in the participating hospitals. Baseline demographic and clinical data consisting of patients’ age, sex, comorbidities (e.g. Hypertension (defined as the systolic blood pressure (SBP) more than 140 mm Hg or the diastolic blood pressure (DBP) more than 90 mm Hg)^17^, hypotension (SBP less than 90 mm Hg or DBP less than 60 mm Hg), Pre- existing CKD (defined as eGFR < 60 ml/min/1.73 m^2^ before AKI diagnosis), diabetes( defined as A1c ≥ 6.5%, or Fasting plasma glucose ≥ 126 mg/dl, or 2-h plasma glucose from an oral glucose tolerance test ≥ 200 mg/dl, or Random plasma glucose ≥ 200 mg/dl), Chronic obstructive pulmonary disease (COPD, defined as a postbronchodilator forced expiratory volume in 1 s (FEV1)/forced vital capacity (FVC) ratio of less than 0.70 and having dyspnea, chronic cough, sputum production, or wheezing symptoms, and a history of exposing to noxious stimuli)^18^, acute myocardial infarction^19^, liver disease (including hepatitis, cirrhosis and so on)^20^, sepsis^21^). Organ failure contains heart failure, respiratory failure, liver failure, central nervous system failure, or gastrointestinal system failure^21^, clinical procedures during hospitalization (e.g. Mechanical ventilation, renal replacement therapy (RRT)), classification, stages and types of AKI. The clinical procedures during hospitalization have been defined in a recent publication^14^. We divided the cause classification of AKI into pre-renal (e.g. hypovolemia), intrinsic-Renal (e.g. nephrotoxic drugs, rhabdomyolysis), post-renal (e.g. urinary tract obstruction) and unclassified AKI. Community-acquired AKI was identified when patients met the KDIGO AKI definition according to serum creatinine change on the first day of admission, or the SCr level was ≥ 1.4 mg/dl in men or ≥ 1.1 mg/dl in women on the first day of admission and ≥ 1.5-fold of the minimal SCr level during hospitalization. Hospital-acquired AKI (HA-AKI) was identified when patients who developed AKI but did not meet community-acquired AKI criteria^22^. The laboratory data included anemia (defined as hemoglobin ≤ 10 g/dl), hypoalbuminemia (defined as serum albumin < 30 g/l), proteinuria (defined as dipstick urinalysis protein positive), and hyperuricemia (defined as serum uric acid ≥ 7.0 mg/dl in men and 6.0 mg/dl in women), which was determined within 24–48 h after AKI diagnosis. SCr was assessed when the patient was discharged from hospital. Renal recovery at discharge has been classified into three levels: (1) Complete renal recovery at discharge was defined as no AKI at discharge, which means the decrease of SCr to the baseline or the physiological range. (2) Partial renal recovery was defined as the decrease of SCr by more than 25% of peak concentration and the levels of SCr that was higher than the physiological range or baseline and without the need for renal replacement therapy at discharge. (3) Failure renal recovery was defined as the decrease of SCr by less than 25% of peak concentration and the levels of SCr that was higher than the physiological range or baseline or remaining dependent on dialysis at discharge^23,24^.

The parameters were regarded as the variable for models to predict the mortality of AKI patients. Missing data of these exposures were imputed by the median for continuous variables and the maximum frequency for categorical variables. All of the data and processes had been collected and analyzed by nephrologists and renal fellows who had completed training.

### Definition of outcomes

The primary clinical outcome was all-cause mortality 30 days or 1 year after AKI diagnosis. These endpoint events were checked by reviewing the hospital’s electronic medical records and the electronic system of Chinese Center for Disease Control and Prevention. We contacted the individual patient or their family member to confirm again by calling or sending a message.

### Identification AKI and baseline creatinine definitions

According to 2012 KDIGO criteria AKI^2^ was characterized by an increase in SCr of more than 0.3 mg/dl (26.5 μmol/l) within 48 h or ≥ 50% from the baseline SCr value within 7 days. AKI has been classified into three stages. AKI stage 1 was defined as an increase in SCr of 0.3 mg/dl (26.5 μmol/l) or more than 50–100% from the baseline SCr value. Stage 2, 3 was defined by more than 100–200%, 200–300% increase in serum creatinine from the baseline SCr value, respectively. Serum creatinine ≥ 4 mg/dl was also regarded as stage 3. Urine output criteria were not used to define AKI in this study.

Baseline SCr was defined as the mean outpatient values between 7 and 365 days before the hospital admission^25^. The mean creatinine concentration during hospital stay before AKI was regarded as the baseline SCr when patients were hospitalized for more than 10 days^25^. If patients have no reliable baseline SCr record and no diagnostic evidence of CKD before admission, we will perform Modification of Diet in Renal disease Trial (MDRD) to estimate the baseline SCr level with the assumption of an eGFR of 75 ml/min/1.73 m^2^^26^.

### Statistical analysis

All data were analyzed using the statistical software SPSS version 18.0 (IBM) and Stata version 14.0. Patients were randomly divided into a test group (70%) by SPSS and then, validated in the remaining 30% of patients (internal validated group). Baseline demographic, clinical and laboratory characteristics were compared between the test group and internal validated group. Data were expressed as the mean ± standard, frequencies, and percentages. The measurement data were compared by Student’s t-test (for normally distributed data) or the Mann–Whitney U test (for non-normally distributed data). The enumeration data used chi-square test. Specifically, AKI stage and renal recovery at discharge were regarded as risk factors for long-term mortality, Kaplan–Meier curves and log-rank were used to reanalyze patient short- and long- survival for the 3 severity levels of renal function at discharge and 3 severity levels of AKI.

In the test group, univariable analysis (chi-square test for enumeration data and Student’s t-test for measurement data) and multivariable logistic regression analyses were performed to assess which factor had a statistically significant effect on mortality and establish two new scoring models for the prediction of mortality within 30-day, 1-year in elderly AKI patients. Variables were included in the multivariate model if they have P < 0.20 in univariate analysis. Variables with 2-tailed P < 0.05 in multivariate analysis were retained in the model. The area under a receiver operating characteristic curve (AUROC) and Hosmer–Lemeshow (H–L) test were used to calculate discrimination and calibration, respectively, to assess the predictive ability of models. The cutoff values, sensitivity, and specificity were calculated by the AUROC analysis. We had selected the cutoff values by the best Youden index. All statistical tests were two-sided. P < 0.05 was considered significant.

### Statement of ethics

The Medical Ethics Committee of the Second Xiangya Hospital of Central South University approved the study protocol and waived the patient consent. This project has been registered in Chinese Clinical Trial Registry (ChiCTR 1800019857).

## Results

### Incidence and baseline characteristics

As shown in Fig. 1, Between January 1st, 2015 and December 31st, 2015, 944 patients were diagnosed with AKI in 8432 hospitalized patients who had more than two serum creatinine tests within 7 days, the overall annual incidence of AKI is approximately 11.79% in hospitalized patients. Among AKI patients, 453 (47.99%), 184 (19.49%), and 307 (32.52%) developed stages 1, 2, and 3 of AKI, respectively. Furthermore, 102 AKI patients were lost to follow-up. Of the 842 patients diagnosed as AKI who be able to follow-up, 589 patients were randomly assigned to the test group, and 253 patients in the internal validation group. The baseline demographic, clinical and laboratory data were described in the Supplementary Table S1, and a comparison between the test group and validation group was performed. Just the incidences of hypercholesterolemia were significantly lower in the test group compared with the validated group. There were no differences in other variables between the groups. The development of AKI stage 3 was 32.43% in the test group (n = 191), and 32.81% in the validation group (n = 83). The AKI severity was associated with the kidney adverse outcome of patients. The rate of patients who were unable to regain renal function at discharge was 42.11% in the test group compared with 44.26% in the validated group.

### Clinical outcomes

Over the course of the study in the test group, 183 (31.07%) AKI patients died within 1 year, with nearly 65.03% (119) of those deaths occurring within the 30 days of the AKI, the all-cause mortality in older AKI patient within 30 days is 20.20%. In the validation group, the mortality within 30-day, 1-year, was 22.31% (55), 30.43% (77), respectively. Early diagnosis and timely treatment for elderly patients to decreasing mortality are important.

### Patient survival analysis

**Figure 2:**
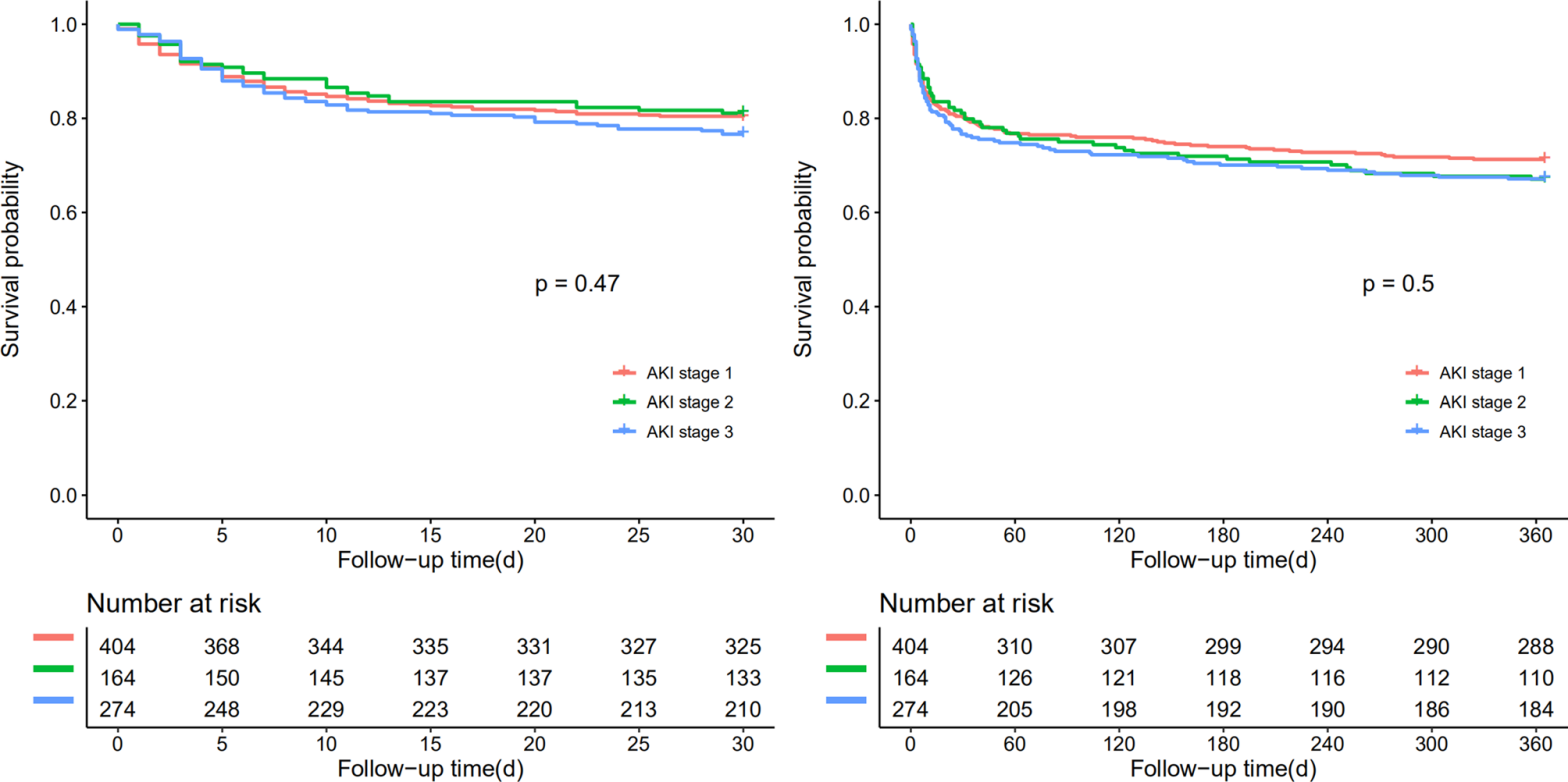
Patient survival within 30 days and 1 year of patients with different stages of AKI. Follow-up time (day): refers to the time since the AKI of diagnosis.

**Figure 3:**
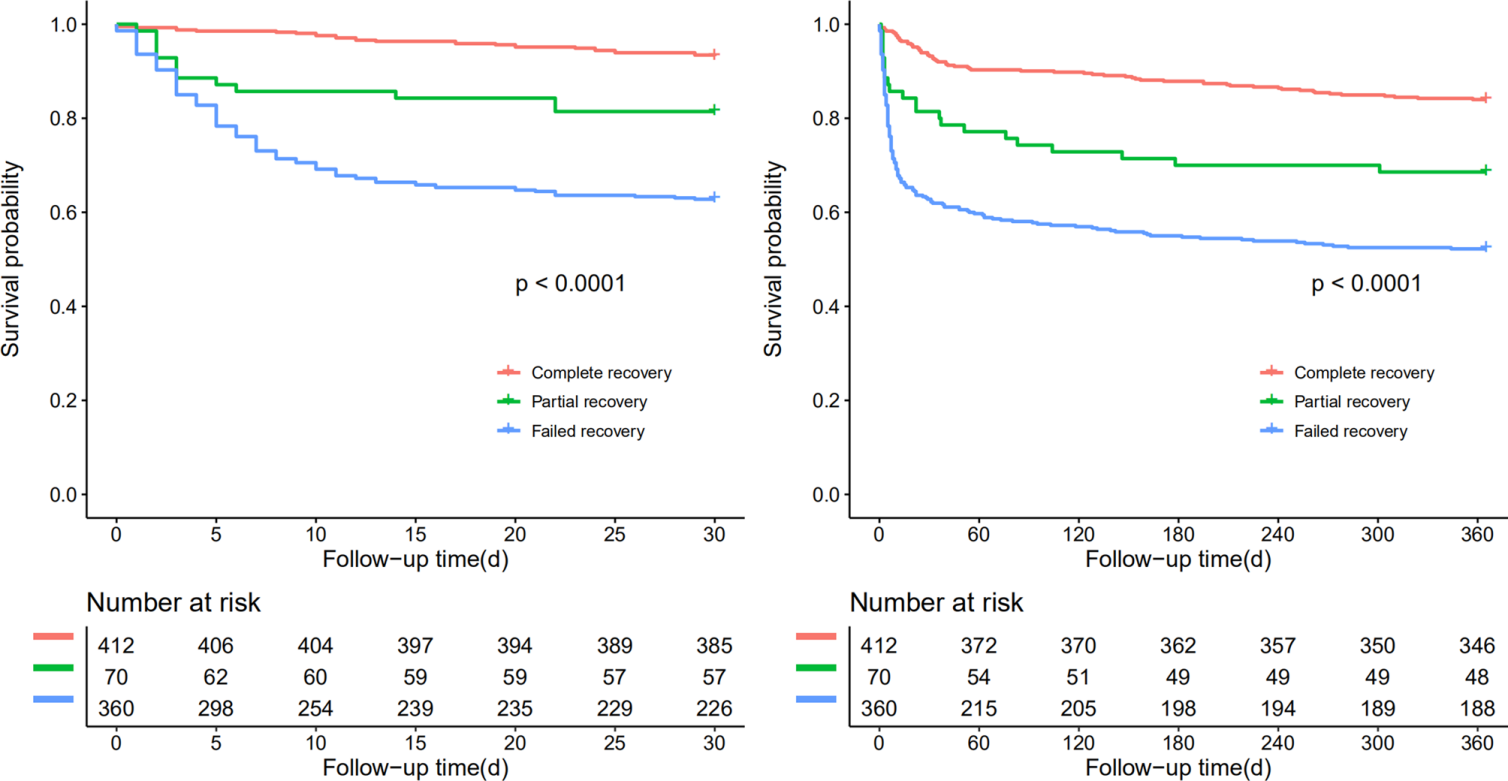
Patient survival within 30 days and 1 year of patients at discharge having different renal function status. Follow-up time (day): refers to the time since the AKI of diagnosis.

Figures 2 and 3 compared the overall survival rate within 30-days, 1-year among the elderly AKI patients with the different status of renal function at discharge, and the different status of AKI stage. The overall survival rate within 30-days, 1-year was significantly higher in the group of completed recovery than in other groups (P < 0. 05), implying that the status of renal function at discharge was associated with the outcome of older AKI patients. However, no significant difference was found in the overall survival rate within 30-days, 1-year among the older AKI patients with the different AKI stages.

### Risk factors of all-cause death within 30 days, 1 year in the older AKI patients

The data of clinic characteristics that were no differences between the test group and validated group was involved in univariate logistic regression analyses, which revealed that AKI type, renal function at discharge, hypertension, acute myocardial infarction, chronic obstructive pulmonary disease, cerebrovascular disease, liver disease, the number of organ failures, anemia and proteinuria were associated with all-cause death within 30 days in the test cohort (Supplementary Table S2) and AKI type, the cause of AKI, solid tumor, renal replacement therapy, acute myocardial infarction, chronic obstructive pulmonary disease, cerebrovascular disease, sepsis, number of organ failures anemia, mechanical ventilation, the number of organ failures, hypoproteinemia and proteinuria were associated with all-cause death within 1 year in the test cohort (Supplementary Table S3). The further multivariate logistic regression analysis showed that AKI type, renal function at discharge, hypotension, acute myocardial infarction, liver disease, the number of organ failures, and proteinuria were independent risk factors that affected the death of patients within 30 days. AKI type, solid tumor, renal replacement therapy, acute myocardial infarction, mechanical ventilation, the number of organ failures, and proteinuria were independent risk factors that affected the death of patients within 1 year.

### Development and validation of all-cause death within 30 days prediction model in elderly hospitalized AKI patients (model 1)

The corresponding integrals of various OR values of the independent risk factors for all-cause death within 30 days after AKI was enrolled in the scoring model. The scoring formula of all-cause mortality within 30 days was as follows: the points of AKI type + the points of renal recovery at discharge + the points of hypotension + the points of acute myocardial infarction + the points of liver disease + the points of the number of organ failures + the points of proteinuria. The scoring criteria were presented in Table 1. The maximum score of points allowed is 24.

**Table 1:** Seven-variable risk index for affecting 30-day mortality in the hospitalized elderly patients with AKI.

	P value	OR (95% CI)	Score
**AKI type**	< 0.001	3.226 (1.866–5.577)	
CA-AKI			0
HA-AKI			3
**Renal function at discharge**			
Complete recovery	0.02		0
Partial recovery	0.26	1.461 (0.756–2.823)	1
Failed recovery	0.004	2.340 (1.312–4.175)	2
**Hypotension**	< 0.001	2.683 (1.555–4.627)	
No			0
Yes			3
**AMI**	0.03	2.228 (1.076–4.616)	
No			0
Yes			2
**Liver disease**	0.02	1.958 (1.107–3.463)	
No			0
Yes			2
**The number of organ failure**			
0			0
1–2	< 0.001	5.34 (2.181–13.073)	5
> 2	< 0.001	9.326 (5.27–20.175)	9
**Proteinuria**	< 0.001	2.771 (1.563–4.912)	
No			0
Yes			3
Maximum	24		

*AKI* acute kidney injury, *CA-AKI* community acquired AKI, *HA-AKI* hospital acquired AKI, *AMI* acute myocardial infarction, *CI* confidence interval, *OR* odds ratio.

The model 1 had a good calibration for predicting the mortality within 30 days in the test group, and the P value of overall goodness-of-fit was > 0.05 (0.857). In the test group, the AUROC was 0.90 (95% CI 0.875–0.932). The AUROC was 0.907 (95% CI 0.865–0.949) in the validation group. The scoring model had high sensitivity and specificity, the sensitivity was 83.40% (test group), 83.00% (validation group) and the specificity was 86.60% (test group), 83.35% (validation group) (Table 3). The results showed that it was reliable to predict the patient who occurred in the all-cause death within 30 days after AKI by model 1.

According to the best Youden index, we found the cutoff value was 11. Risk stratification was conducted for all patients: low-risk group (0–10) and high-risk group (≥ 11). The all-cause mortality within 30 days was significantly higher in the high-risk group than in the low-risk group (Supplementary Table S4).

### Development and validation of all-cause death within 1 year prediction model in elderly hospitalized AKI patients (model 2)

We assigned various OR values to the independent risk factors for all-cause death within 1 year after AKI. The scoring formula was as follows: the points of AKI type + the points of solid tumor + the points of renal replacement therapy + the points of acute myocardial infarction + the points of mechanical ventilation + the points of the number of organ failures + the points of proteinuria. The scoring criteria were presented in Table 2. The maximum score of points allowed is 25.

**Table 2:** Seven-variable risk index for affecting 1-year mortality in the hospitalized elderly patients with AKI.

	P value	OR (95% CI)	Score
**AKI type**	< 0.001	2.540 (1.587–4.067)	
CA-AKI			0
HA-AKI			3
**Malignancy**			
No			0
Non-metastasis	0.003	2.316 (1.328–4.039)	2
Metastasis	0.003	4.347 (1.667–11.335)	4
**RRT**	0.01	3.456 (1.323–9.027)	
No			0
Yes			3
**AMI**	0.01	2.369 (1.216–4.616)	
No			0
Yes			2
**Mechanical ventilation**	0.002	2.284 (1.368–3.813)	
No			0
Yes			2
**The number of organ failure**			
0			0
1–2	< 0.001	3.049 (1.660–5.602)	3
> 2	< 0.001	7.906 (3.662–17.065)	8
**Proteinuria**	< 0.001	2.972 (1.769–4.993)	
No			0
Yes			3
Maximum	25		

*AKI* acute kidney injury, *CA-AKI* community acquired AKI, *HA-AKI* hospital acquired AKI, *AMI* acute myocardial infarction, *RRT* renal replacement therapy, *CI* confidence interval, *OR* odds ratio.

By using the Hosmer–Lemeshow (H–L) test to assess the calibration of the model 2, the result showed a high goodness-of-fit. The area under ROC curve of 1-year mortality was favorable, In the test group, the AUROC of 1-year mortality in the test and validation group was 0.853 (95% CI 0.818–0.887) and 0.851 (95% CI 0.797–0.905), respectively. They also had high sensitivity (77.10%, 76.10%) and specificity (82.40%, 80.02%) both in the test and validation group (Table 3).

**Table 3:** Predictive performance of prediction model about elderly AKI patients within 30-day and 1-year all-cause death in the test and validation groups.

	Goodness of fit (P value)	AUROC ± SD (95% CI)	Sensitivity (%)	Specificity (%)	Youden index	Cut-off value
**Model 1**						
Test group (N = 589)	3.998 (0.86)	0.903 ± 0.015 (0.875–0.932)	83.40	86.60	0.701	11 (10.5)
Validation group (N = 253)	–	0.907 ± 0.021 (0.865–0.949)	83.00	83.50	0.665	11 (10.5)
**Model 2**						
Test group (N = 589)	3.466 (0.81)	0.853 ± 0.018 (0.818–0.887)	77.10	82.40	0.595	9 (8.5)
Validation group (N = 253)	–	0.851 ± 0.027 (0.797–0.905)	76.10	80.20	0.563	9 (8.5)

*AUROC* the area under the receiver operating characteristic curves, *SD* standard deviation, *CI* confidence interval.

According to the best Youden index, the cutoff value was 9, all patients were conducted risk stratification: low-risk group (0–8) and high-risk group (≥ 9), the all-cause mortality within 1-year was significantly higher in the high-risk group than in the low-risk group (Supplementary Table S5).

### Comparison of new scoring models (model 1, model 2), SOFA, and APACHE II in predicting all-cause mortality within 30 days, 1 year after acute kidney injury

To assess the accuracy of the models in predicting outcome, we compared the representative scoring systems with our models in the sensitivity and specificity. The sensitivity and specificity of model 1 were superior to SOFA and APACHE II in predicting the mortality of older AKI patients within 30 days in the test group (Sensitivity: 83.40% vs 76.10%, 63.20%; Specificity: 86.60% vs 68.10%, 82.30%, respectively), the similar results were presented in the validation group. Whatever it is in the test group or validation group, the AUROC of model 2 was better than SOFA and APACHE II (Supplementary Table S6).

## Discussion

Age was an independent risk factor for the initiation, development, and adverse outcome of AKI^27,28^. This study represented the analysis of AKI among hospitalized elderly patients in China. The incidence and mortality of AKI among hospitalized elderly patients were hard to be compared in different countries and centers due to a not generally accepted definition of the elderly and the lack of a gold standard for identification of AKI^11,29^. In this study, the incidence of AKI in elderly patients (age ≥ 65 years) was 11.79%. A report from China showed that the incidence of AKI in 65–80 years old patients was 15.44% and in the very older age group (age > 80 years) was 22.22%^30^, which was higher than our study because a novel approach of the baseline SCr (a baseline SCr was defined as the mean of SCr levels within the 7 days before any time points, the SCr data within 7 days after that time point was compared with this baseline) was used in their research.

The short-term mortality of older AKI patients varied from 10 to 75% as reported in various studies^30–32^. Our study has shown that the 30-day mortality rate of elderly AKI inpatients was 19.35%, and the 1-year was 30.22%. Because of variables among different studies, the mortality of elderly AKI patients was quite different. The results presented that the stage of AKI was not associated with the all-cause death of the elderly patients within 30 days, 1 year. That differed from the previous conclusion from the adult AKI patients^23^. One of the distinct features of these older adults was that they have several severe comorbidities than younger, the chief cause of death may be the cumulative effect of other comorbidities rather than the damage of renal function^31,33^. For example, many older adults develop AKI as a complication of non-kidney disease (such as coronary heart disease)^29^.

Effective studies of the risk factors for the mortality of AKI patients have been investigated. The common predictors of AKI patient death were the stage of AKI, failed organ systems, sepsis, the duration of ICU stay, preexisting CKD, and oliguria occurrence at AKI diagnosis^34–36^ and some researches reported that a slight increase in serum creatinine during admission has been associated with patient death^37^. However, most publications have investigated prognostic risk factors in adult AKI patients, only a few studies focused on elderly patients. With the increasing of age, patients show poorer renal foundation condition and recuperative powers, the risk factors of mortality in the older adults may be different from the past results from the adults. A study showed that age, cerebrovascular disease, cancer, and mechanical ventilation affected the mortality of Chinese older AKI patients^16^. In our study, the age of the research subject was more than 65, we found that the type of AKI, the number of failed organ systems, proteinuria, and acute myocardial infarction were both the short-term and long-term all-cause death independent risk factors. We can see that from the older adults, the multiple comorbidities were the risk factors for the all-cause death, while the decrease of renal function has much more sway over the mortality of adult AKI patients. We speculate that such difference between the elderly and adults was due to the following reason. Tubular function deficits result in volume overload, hyperkalemia, encephalopathy, and pericarditis^38^. Elderly AKI patients have a limited capacity of body recovery. In this condition, The elderly with comorbidities have more chance to obtain multiple organ failure and the highest mortality. AKI type was also a risk factor affected elderly AKI patient’ outcome. The short-term and long-term all-cause mortality associated with HA-AKI was higher compared with CA-AKI, indicating a poorer prognosis. That was similar to the previous meta-analysis which included fifteen eligible studies involving 46,157 patients^39^. A plausible explanation for an unfavorable prognosis in patients with HA-AKI may be that HA-AKI patients lack timely treatment by nephrologists because HA-AKI may occur in every department in the hospital and CA-AKI patients are often admitted to the nephrology department^39^. Two other factors (the number of organ failure and proteinuria) are also affecting both the short-term and long-term all-cause mortality. Many studies reports demonstrated that proteinuria is a well-established biomarker for predicting AKI developing to CKD^40,41^. However, proteinuria was also a potentially modifiable risk factor in cardiovascular events and mortality. Glomerular capillary permeability and tissue inflammation may result in proteinuria, proteinuria was related to tissue damage and the systemic inflammatory response to acute insult^41,42^.

In this study, we demonstrated that two new scoring models (model 1, model 2) predicted the development of all-cause death within 30-day, 1-year, respectively. Two new scoring models were mostly composed of the clinical manifestations and laboratory examinations at the time of AKI diagnosis. These models were used to semi-quantify the risk factors of all-cause death in AKI patients. In model 1, we focused on the death risk assessment within 30 days after AKI. We obtained the cut-off value (11 points) from the test group and found that when the total score of model 1 was < 11, ≥ 11, the mortality rate was 6.62%, 20.54%, respectively. The AKI patient whose score of the model 1 is ≥ 11 will have more chance to develop all-cause death within 30 days after AKI diagnosis. The analysis was made to verify the results in the validation group, which showed that the risk stratification model had good stability and repeatability. This scoring model seemed to be a practical and available tool for predicting the short-term death risk for older AKI patients. We also performed an analysis in patients with the total score of the model 2 < 9 to determine the performance of the model 2 in the prediction of the older AKI patients’ all-cause death within 1 year. In this part of patients, model 2 continued to perform well and the mortality of those patients was lower than the total score of ≥ 9, which indicated that the model 2 can be more effectively and quickly in predicting the long-term outcome of elderly AKI patients by clinical indicators. No good predicting mortality model for the elderly is available, and the clinicians mainly judge elderly AKI patients’ outcome on the change of serum creatinine concentration. However, in the present study about the elderly AKI patients, we used two statistical methods for analyzing the data found that the AKI stage was not a risk factor for short-term and long long-term mortality. The clinicians should pay more attention to the elderly AKI patients with comorbidity, because they have a high risk of developing organ failure and death. But only considering one characteristic of AKI patients is incomplete for predicting the outcome, many risk factors can affect the prognosis of AKI. We developed two models to calculate the risk of short-term and long-term death, making clinicians instantly and effectively recognize AKI patients with a high risk of progressing to death. Our study also provides information about clinical individualized treatment, and improve the prognosis of elderly AKI patients.

The representative prognostic scoring systems (SOFA, APACHE II) were also used to assess the prognosis of AKI patients and compared it with our scoring models in the sensitivity and specificity about short-term and long-term death. Our scoring models presented better discrimination and gave better predictions. One explanation was that the older scoring systems were derived from ICU, and our models may suit older AKI patients.

However, there were some limitations in this study. First, this was a single-province multi-center study, which affected the overall representation of the study population and the external validity of the research results. Second, only the serum creatinine was the diagnostic criterion of AKI, and the change of urine output meeting the 2012 KDIGO criteria were not defined as AKI. This study also underestimated the overall incidence of AKI in the elderly. But some researchers suggested that using urine output in 2012 KDIGO criteria to define AKI may be liberal^43^. Thirdly, data collection is retrospective, leading to recall and selection bias. National, multi-center prospective trials about our prognostic scoring models of hospitalized elderly AKI needed to be conducted, which will be a good test to verify the accuracy and validity of models.

## Conclusion

In summary, in a cohort of hospitalized elderly AKI patients in China, our study found an 11.78% incidence of AKI, described the risk factors of all-cause death within 30 days, 1 year, and developed two new scoring models to predict mortality of short- and long-term. These models in this study semi-quantifying the association between AKI outcome and the risk factors for clinicians can predict the outcome of hospitalized elderly AKI patients in the early stage, give a reasonable personalized therapeutic scheme and obtain a better clinical prognosis.

## Supplementary Information

Supplementary Information.

## Data Availability

The datasets were analysed during the current study available from the corresponding author on reasonable request.
