# Supplementary material for: Development of mortality prediction model in the elderly hospitalized AKI patients
Source: Sci Rep. 2021 Jul 26;11:15157. doi: 10.1038/s41598-021-94271-9 (PMC8313696; doi:10.1038/s41598-021-94271-9)
Supplement: Supplementary file 1 — Supplementary Information. [file 41598_2021_94271_MOESM1_ESM.pdf]

Supplementary Table 1. Baseline Demographic and Clinical Parameters of AKI Patients in the Test and Validation Group

variable	Group		P Value*	95% CI
	Test group (n=589)	Validation group (n=253)		
sex (men), No. (%)	389(66.04%)	161(63.64%)	0.48	(0.661-1.224)
**AKI type, No. (%)**			0.81	(0.776-1.411)
CA-AKI	238(40.41%)	105(41.50%)		
HA-AKI	351(59.59%)	148(58.50%)		
**AKI stage, No. (%)**			0.88	/
1	286(48.56%)	118(46.64%)		
2	112(19.02%)	52(20.55%)		
3	191(32.43%)	83(32.81%)		
**AKI classification, No. (%)**			0.15	/
Pre-renal	415(70.46%)	163(64.43%)		
Intrinsic-Renal	105(17.83%)	48(18.97%)		
Post-renal	42(7.13%)	22(8.70%)		
Unclassified	27(4.58%)	20(7.90%)		
**AKI comorbidity, No. (%)**				
hypotension	145(24.62%)	54(21.34%)	0.31	(0.844-1.715)
hypertension	327(55.52%)	143(56.52%)	0.79	(0.713-1.292)
Pre- existing CKD (eGFR < 60 ml/ min/1.73 m^2)	139(23.60%)	65(25.69%)	0.59	(0.636-1.255)
Diabetes	162(27.50%)	56(22.13%)	0.08	(0.943-1.889)
AMI	64(10.87%)	20(7.91%)	0.19	(0.840-2.401)
COPD	67(11.38%)	22(8.70%)	0.28	(0.813-2.235)
Cerebrovascular Disease	130(22.07%)	67(26.48%)	0.16	(0.559-1.105)
Liver disease	111(18.85%)	62(24.51%)	0.07	(0.503-1.108)
Solid tumor ， non-metastasis	105(17.83%)	54(21.34%)	0.07	(0.523-1.028)
Solid tumor , metastasis	24(4.07%)	16(6.32%)	0.16	(0.328-1.206)
Sepsis	75(12.73%)	25(9.88%)	0.25	(0.824-2.148)
**Organ failure, No. (%)**				
Heart	189(32.09%)	75(29.64%)	0.43	(0.764-1.732)
Respiratory	147(24.96%)	63(24.90%)	0.99	(0.899-1.789)
Central nervous system	166(28.18%)	56(22.13%)	0.07	(0.976-1.953)
Gastrointestinal system	58(9.85%)	25(9.88%)	0.92	(0.596-1.603)
liver	158(26.83%)	61(24.11%)	0.42	(0.821-1.623)
**Renal function at discharge , No. (%)**			0.86	/
Complete recovery	293(49.74%)	119(47.04%)		
Partial recovery	48(8.15%)	22(8.70%)		
Failed recovery	248(42.11%)	112(44.26%)		
**Clinical Procedures, No. (%)**				
RRT	36(6.11%)	22(8.70%)	0.17	(0.747-1.718)
Cardic surgon	23(3.90%)	4(1.58%)	0.08	(0.869-7.417)
Mechanical ventilation	166(28.18%)	61(24.11%)	0.24	(0.875-1.726)

variable	Group		P Value*	95% CI
	Test group (n=589)	Validation group (n=253)		
**Clinical Parameter**				
anemia, No. (%)	210(35.65%)	85(33.60%)	0.58	(0.803-1.494)
proteinuria, No. (%)	117(19.86%)	36(14.23%)	0.06	(0.995-2.244)
hyperuricemia, No. (%)	242(41.09%)	119(47.04%)	0.10	(0.584-1.056)
hypoalbuminemia, No. (%)	185(31.41%)	79(31.23%)	0.99	(0.734-1.386)
hypercholesterolemia, No. (%)	37(6.28%)	21(8.3%)	0.05	(0.786-1.325)
hyperkalemia, No. (%)	74(12.56%)	30(11.86%)	0.78	(0.679-1.679)
BUN, mmol/L	16.17±1.72	13.14±0.58	0.09	/
peak of SCr, mmol/L	282.30±9.94	284.65±15.43	0.90	/

AKI = Acut Kidney Injure ; CA-AKI = Community Acquired AKI; HA-AKI = Hospital Acquired AKI; CKD = Chronic Kidney Disease ; eGFR =Estimated Glomerular Filtration Rate ; AMI = Acute myocardial infarction; COPD = Chronic Obstructive Pulmonary Disease; RRT = Replacement Renal Treatment; BUN = Blood Urea Nitrogen; SCr= Serum Creatinine; No=Number；CI = confidence interval.

Supplementary Table 2. Univariable Analysis of All-Cause Death within 30 days in theTest Group

variable	Group		P Value*	95% CI
	Survival group (n=475)	Death group (n=114)		
sex (men), No. (%)	174(36.63%)	37(32.46%)	0.16	(0.559-1.215)
**AKI types, No. (%)**			0.004	(1.032-2.390)
CA-AKI	207(43.58%)	40(35.09%)		
HA-AKI	268(56.42%)	74(64.91%)		
**AKI stage, No. (%)**			0.47	/
1	222(46.74%)	55(48.25%)		
2	99(20.84%)	18(15.79%)		
3	154(32.42)	41(35.96%)		
**AKI classification, No. (%)**			0.04	/
Pre-renal	330(69.47%)	84(73.68%)		
Intrinsic-Renal	84(17.68%)	16(14.04%)		
Post-renal	39(8.21%)	10(8.77%)		
Unclassified	22(4.63%)	4(3.51%)		
**AKI comorbidity, No. (%)**				
hypotension	271(57.05%)	61(53.51%)	0.20	(0.575-1.306)
hypertension	94(19.79%)	39(34.21%)	＜0.001	(1.109-4.032)
Pre- existing CKD (eGFR < 60 ml/ min/1.73 m²)	112(23.58%)	28(24.56%)	0.83	(0.856-1.273)
Diabetes	123(25.89%)	39(34.21%)	0.15	(0.841-1.862)
AMI	38(8.00%)	21(18.42%)	＜0.001	(2.568-5.489)
COPD	50(10.53%)	10(8.77%)	0.01	(1.370-3.613)
Cerebrovascular Disease	106(22.32%)	27(23.68%)	0.05	(1.262-2.925)
Liver disease	92(19.37%)	27(23.68%)	0.01	(1.116-2.676)
Solid tumor ，non-metastasis	120(25.26%)	23(20.18%)	0.79	(0.909-1.083)
Solid tumor metastasis	21(4.42%)	3(2.63%)	0.33	(0.560-2.359)
Sepsis	55(11.58%)	16(14.04%)	0.06	(0.685-2.268)
**Organ failure, No. (%)**				
Heart	141(29.68%)	40(35.09%)	＜0.001	(2.904-4.445)
Respiratory	95(20.00%)	47(41.23%)	＜0.001	(1.978-3.065)
Central nervous system	105(22.11%)	41(35.96%)	＜0.001	(6.628-15.265)
Gastrointestinal system	39(8.21%)	18(15.79%)	＜0.001	(2.805-6.356)
liver	114(24.00%)	40(35.09%)	＜0.001	(2.142-4.671)
**Renal function at discharge , No. (%)**			＜0.001	/
Complete recovery	274(57.68%)	19(16.67%)		

variable	Group		P<br>Value*	95% CI
	Survival group (n=475)	Death group (n=114)		
Partial recovery	42(8.84%)	6(5.26%)		
Failed recovery	159(33.47%)	89(78.07%)		
**Clinical Procedures, No. (%)**				
RRT	26(5.47%)	10(8.77%)	0.24	(0.985-2.265)
Cardic surgon	16(3.37%)	3(2.63%)	0.67	(0.542-2.298)
Mechanical ventilation	125(26.32%)	41(35.96%)	0.05	(2.013-3.132)
**Clinical Parameter**				
anemia, No. (%)	158(33.26%)	39(34.21%)	0.002	(1.035-2.173)
proteinuria, No. (%)	68(14.32%)	33(28.95%)	＜0.001	(1.984-4.616)
hyperuricemia, No. (%)	192(40.42%)	50(43.86%)	0.82	(0.655-1.368)
hypoalbuminemia, No. (%)	150(31.58%)	34(29.82%)	0.47	(0.908-1.259)
hyperkalemia, No. (%)	59(12.42%)	14(12.28%)	0.99	(0.693-1.257)
BUN, mmol/L	14.31±0.45	15.23±0.94	0.37	/
peak of SCr, mmol/L	287.96±11.54	259.96±18.48	0.26	/

Note: AKI = Acut Kidney Injure ; CA-AKI = Community Acquired AKI; HA-AKI = Hospital Acquired AKI; CKD = Chronic Kidney Disease ; eGFR =Estimated Glomerular Filtration Rate; AMI:Acute myocardial infarction; COPD = Chronic Obstructive Pulmonary Disease; RRT = Replacement Renal Treatment; BUN = Blood Urea Nitrogen; Cr= Serum Creatinine; No=Number; CI = confidence interval.

Supplementary Table 3. Univariable Analysis of All-Cause Death within 1 year in the Test Group

variable	Group		P Value*	95%CI
	Survival group (n=411)	Death group (n=178)		
sex (men), No. (%)	272(66.18%)	117(65.73%)	0.92	(0.704-1.478)
**AKI types, No. (%)**			0.001	(1.280-2.706)
CA-AKI	184(44.77%)	54(30.34%)		
HA-AKI	227(55.23%)	124(69.66%)		
**AKI stage, No. (%)**			0.09	/
1	214(52.07%)	72(40.45%)		
2	82(19.95%)	30(16.85%)		
3	115(27.98%)	76(42.70%)		
**AKI classification, No. (%)**			0.03	/
Pre-renal	285(69.34%)	130(73.03%)		
Intrinsic-Renal	77(18.73%)	28(15.73%)		
Post-renal	35(8.52%)	7(3.93%)		
Unclassified	14(3.41%)	13(7.30%)		
**AKI comorbidity, No. (%)**				
hypotension	96(23.36%)	49(27.53%)	0.42	(0.835-1.860)
hypertension	226(54.99%)	101(56.74%)	0.72	(0.749-1.523)
Pre- existing CKD (eGFR < 60 ml/ min/1.73 m²)	100(24.33%)	39(21.91%)	0.52	(0.571-1.325)
Diabetes	111(27.01%)	51(28.65%)	0.69	(0.734-1.605)
AMI	27(6.57%)	37(20.79%)	＜0.001	(2.192-6.355)
COPD	35(8.52%)	32(17.98%)	0.002	(1.356-3.836)
Cerebrovascular Disease	79(19.22%)	51(28.65%)	0.01	(1.123-2.536)
Liver disease	73(17.76%)	38(21.35%)	0.31	(0.801-1.949)
Solid tumor ，non-metastasis	70(17.03%)	35(19.66%)	0.13	(0.911-2.081)
Solid tumor ，metastasis	13(3.16%)	11(6.18%)	0.09	(0.885-4.593)
Sepsis	30(7.30%)	45(25.28%)	＜0.001	(2.600-7.101)
**Organ failure, No. (%)**				
Heart	108(26.28%)	81(45.51%)	＜0.001	(1.616-3.373)
Respiratory	55(13.38%)	92(51.69%)	＜0.001	(1.987-6.686)
Central nervous system	64(15.57%)	102(57.30%)	＜0.001	(4.468-10.815)
Gastrointestinal system	24(5.84%)	33(18.54%)	＜0.001	(2.092-6.404)
liver	89(21.65%)	69(38.76%)	＜0.001	(1.558-3.346)
**Renal function at discharge , No. (%)**			＜0.001	/
Complete recovery	247(60.10%)	48(26.97%)		

variable	Group		P Value*	95%CI
	Survival group (n=411)	Death group (n=178)		
Partial recovery	27(6.57%)	20(11.24%)		
Failed recovery	137(33.33%)	110(61.79%)		
**Clinical Procedures, No. (%)**				
RRT	8(1.95%)	28(15.73%)	＜0.001	(1.600-4.536)
Cardic surgon	19(4.62%)	4(2.25%)	0.17	(0.159-1.415)
Mechanical ventilation	72(17.52%)	94(52.81%)	＜0.001	(3.560-7.751)
**Clinical Parameter**				
anemia, No. (%)	143(34.79%)	67(37.64%)	0.52	(0.782-1.623)
proteinuria, No. (%)	58(14.11%)	59(33.15%)	＜0.001	(1.987-4.582)
hyperuricemia, No. (%)	160(38.93%)	89(50.00%)	0.01	(1.101-2.236)
hypoalbuminemia, No. (%)	119(28.95%)	66(37.08%)	0.05	(1.005-2.115)
hyperkalemia, No. (%)	51(12.41%)	22(12.36%)	0.99	(0.584-1.698)
BUN, mmol/L	14.20±0.48	15.14±0.78	0.29	/
peak of Scr, mmol/L	287.11±12.49	271.69±15.99	0.47	/

Note: AKI = Acut Kidney Injure ; CA-AKI = Community Acquired AKI; HA-AKI = Hospital Acquired AKI; CKD = Chronic Kidney Disease ; eGFR =Estimated Glomerular Filtration Rate; AMI:Acute myocardial infarction; COPD = Chronic Obstructive Pulmonary Disease; RRT = Replacement Renal Treatment; BUN = Blood Urea Nitrogen; Scr= Serum Creatinine; No=Number; CI = confidence interval.

Supplementary Table 4. The mortality of different risk stratification within 30 days after AKI diagnosis

Score	Group , N(%)	
	Test Group N=589	Validation Group N=253
0-10 points	39(6.62%)	17(6.71%)
≥11 points	121(20.54%)	39(15.42%)

Supplementary Table 5. The mortality of different risk stratification within 1 year after AKI diagnosis

Score	Group , N(%)	
	Test Group N=589	Validation Group N=253
0-8 points	52(8.83%)	24(9.49%)
≥9 points	126(21.39%)	43(17.00%)

Supplementary Table 6. Comparison of model 1, SOFA, and APACHE II in predicting 30 days mortality after AKI diagnosis

	AUROC	95% CI for AUROC	Sensitivity	Specificity	Youden Index	Cut-Off Value
**Test Group**						
Model1	0.903	0.875-0.932	83.40%	86.60%	0.700	11（10.5）
SOFA	0.792	0.753-0.832	76.10%	68.10%	0.442	6（5.5）
APACHE II	0.799	0.757-0.840	63.20%	82.30%	0.455	26 (25.5)
**Validation Group**						
Model 1	0.907	0.865-0.949	83.00%	83.50%	0.665	11（10.5）
SOFA	0.832	0.769-0.894	73.60%	75.50%	0.491	6（5.5）
APACHE II	0.848	0.783-0.913	69.50%	89.00%	0.585	26 (25.5)

Note: AUROC = the area under the receiver operating characteristic (AUROC) curves；CI = confidence interval;APACHE II= Acute Physiology and Chronic Health Evaluation II; SOFA=Sequential Organ Failure Assessment.
